# Supplementary material for: DNA methylation signatures of illicit drug injection and hepatitis C are associated with HIV frailty
Source: Nat Commun. 2017 Dec 21;8:2243. doi: 10.1038/s41467-017-02326-1 (PMC5740109; doi:10.1038/s41467-017-02326-1)
Supplement: Supplementary file 1 — Supplementary Information [file 41467_2017_2326_MOESM1_ESM.pdf]

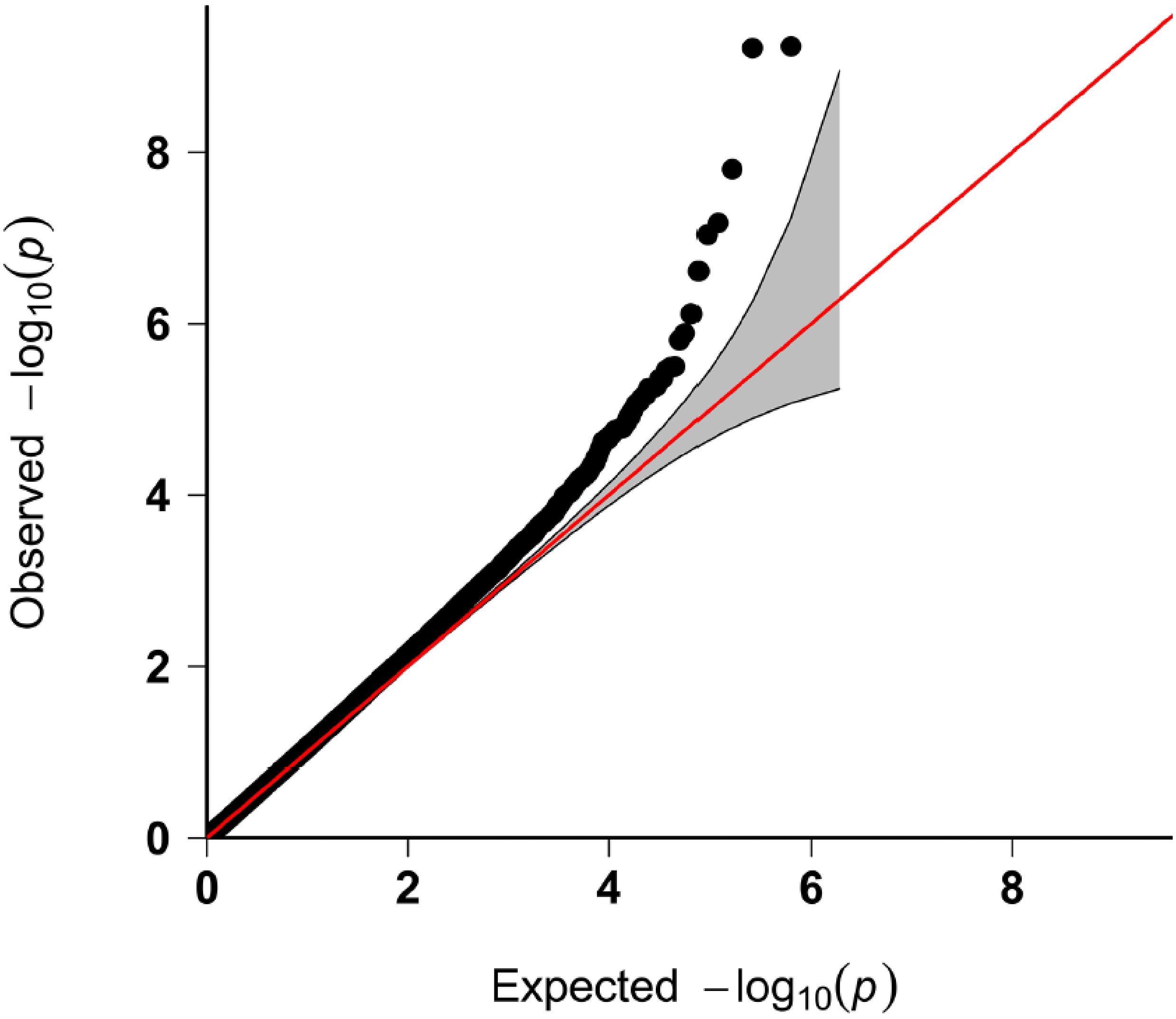

Supplementary Figure 1: A QQ plot showing observed versus expected −log10(p values) for association at all 437,722 CpG sites. λ=1.08 in the discovery epigenome-wide association analysis.

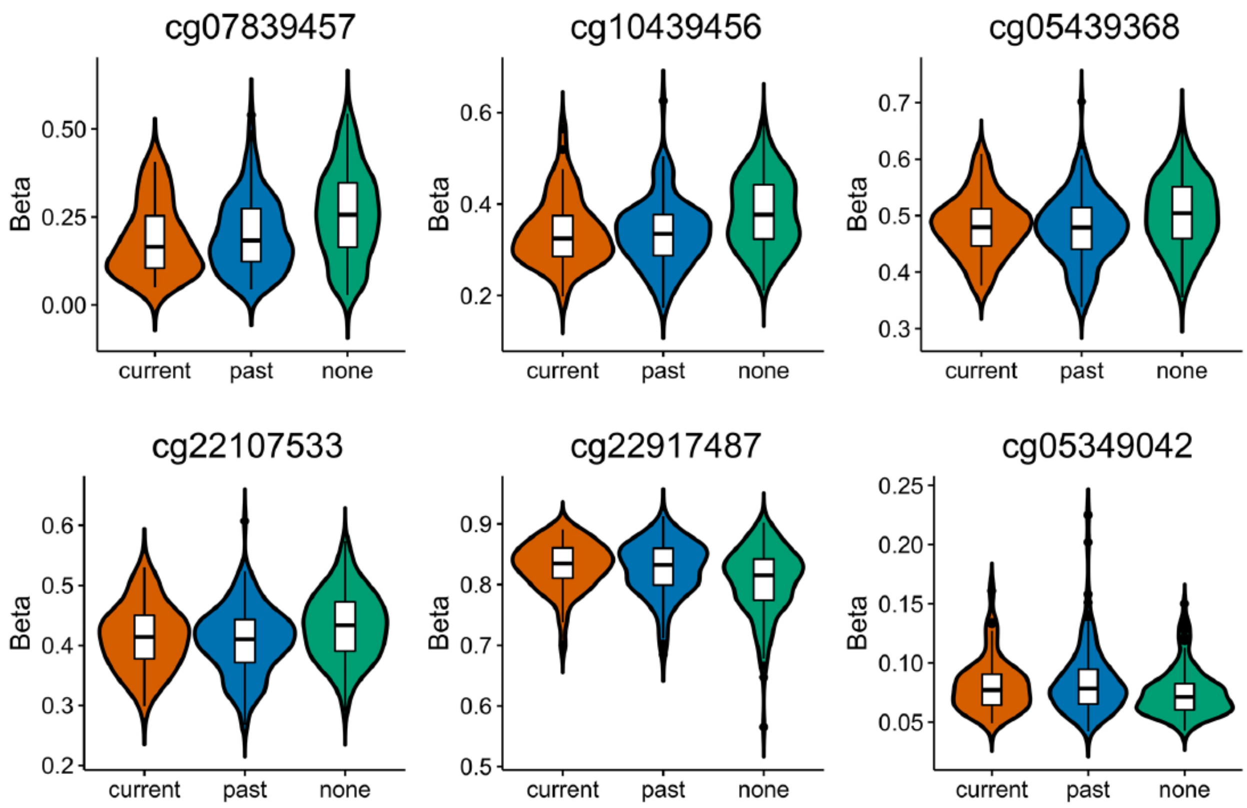

Supplementary Figure 2: Comparisons of DNA methylation at six significant CpG sites among HIV-infected current injection drug use comorbid for hepatitis C (IDU+/HCV+, N=58); past IDU+/HCV+ (N=158), and non-IDU/non-HCV (N=170) in the discovery sample.

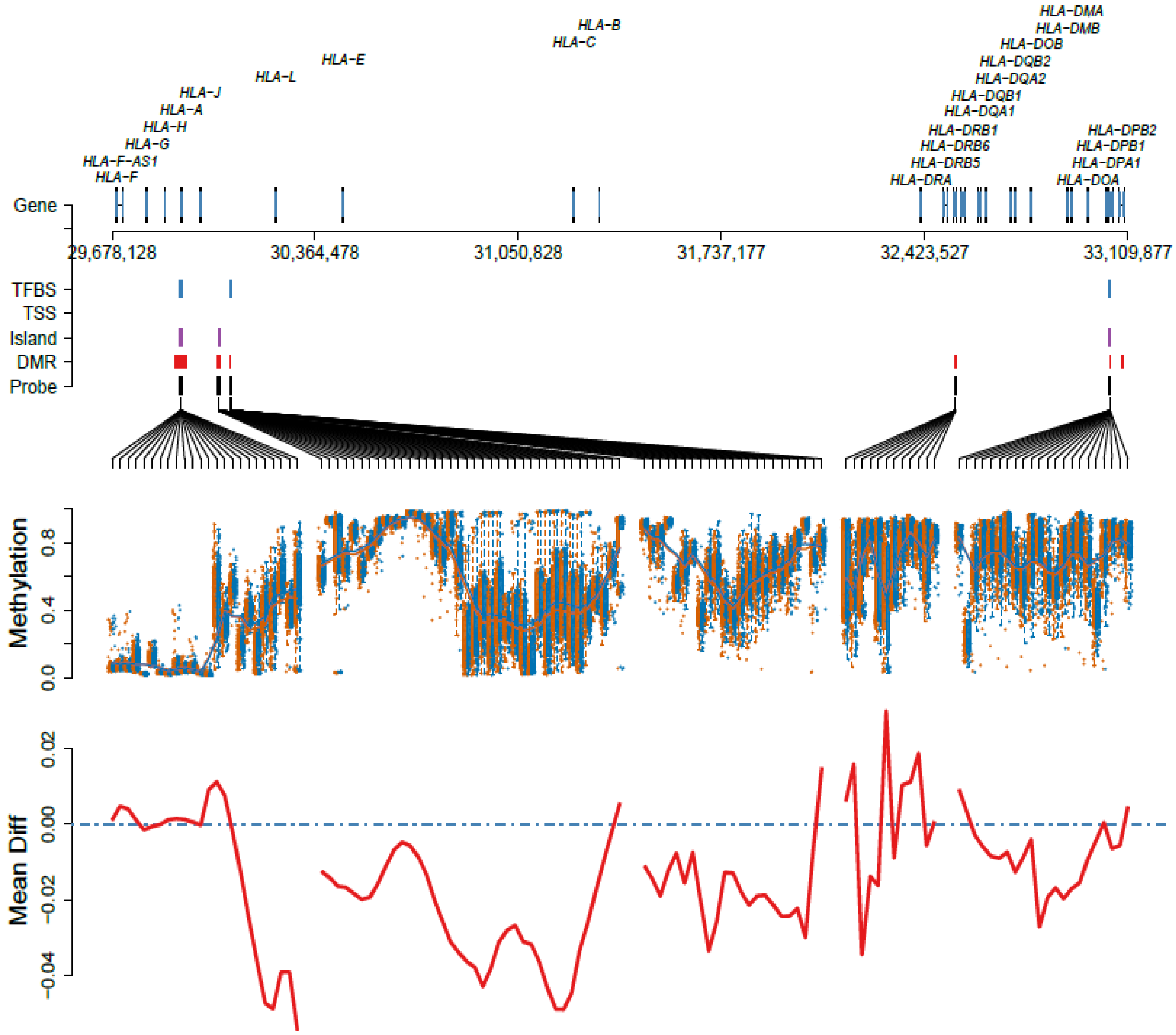

Supplementary Figure 3: Replication of differential methylation region on chromosome 6q21 that hosts MHC class I and II: Top panel: from top to bottom, the panel shows gene location, transcription factor binding sites (TFBS), probes located within 1500 bp or 200 bp from transcription start site (TSS), probes in CpG islands (Island), differential methylation region (DMR), genomic location of each probe. Middle panel: methylation (β) in injection drug use with HCV (IDU+/HCV+) and non-injection drug use without HCV infection (IDU-/HCV-). Bottom

panel: average methylation differences between IDU+/HCV+ and IDU-/HCV-. The replication sample included 104 IDU+/HCV+ and 308 IDU-/HCV- individuals.

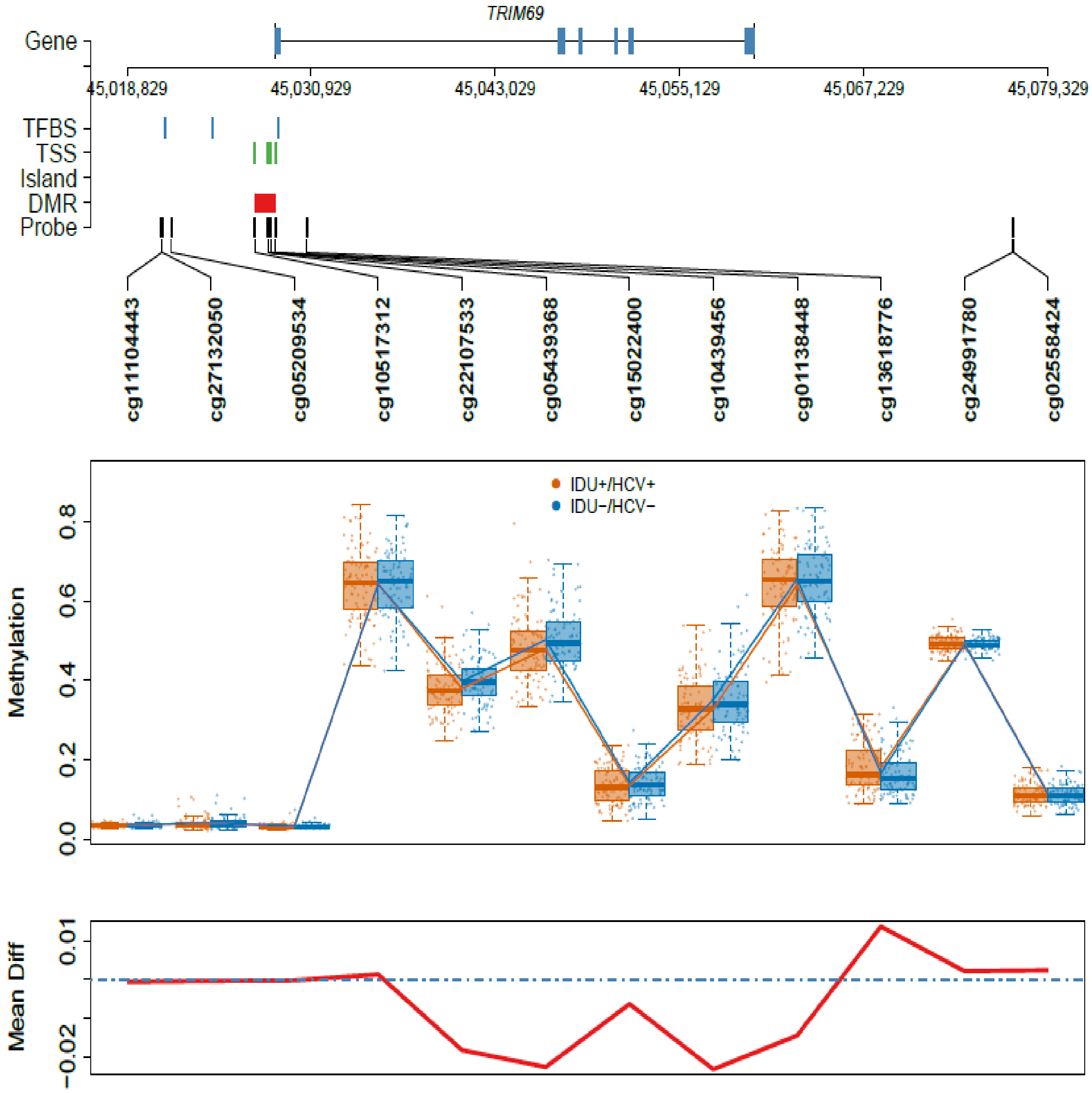

Supplementary Figure 4: Replication of differential methylation region (DMR) on the promoter of *TRIM69* in the discovery sample. Top panel: Genomic location of each probe, transcription binding site, DMR; Middle panel: methylation at each probe in injection drug use with HCV (IDU+/HCV+) and non-injection drug use without HCV (IDU-/HCV-); Bottom panel: average methylation difference between IDU+/HCV+ and IDU-/HCV-. The replication sample included 104 IDU+/HCV+ and 308 IDU-/HCV- individuals.

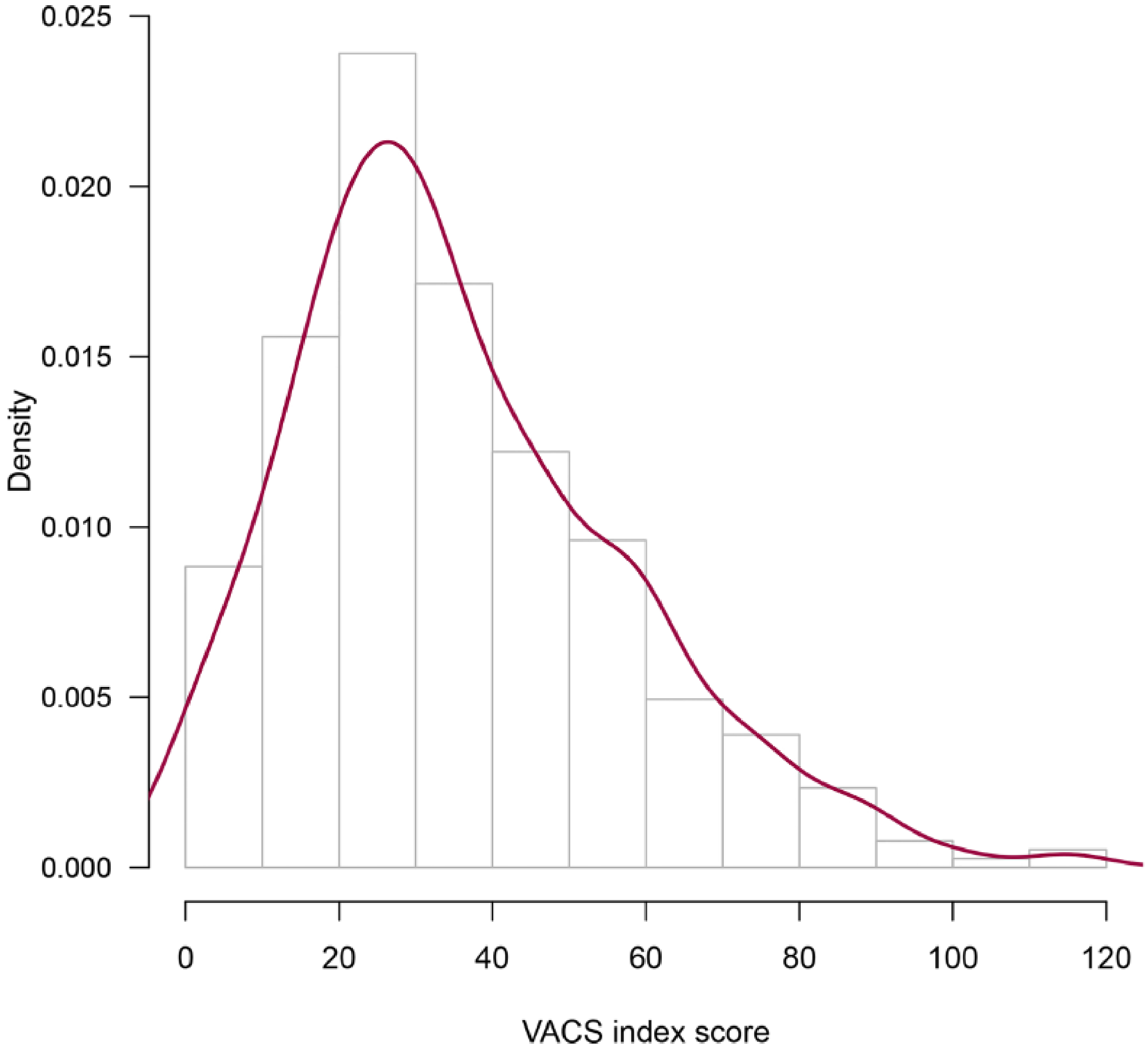

Supplementary Figure 5: Distribution of Veteran Aging Cohort Study indexes (VACS index) among 386 HIV-infected samples in the discovery set. The greater VACS index indicates higher HIV frailty.

Supplemental Table 1 Differential DNA methylation regions between IDU+/HCV+ and IDU-/HCV- groups

Discovery sample

CHR	Start	End	# CpG	p.value	FDR	Near Gene
chr6	29911494	29911558	3	0.001	0.025	***HLA-A***
chr6	28601312	28601419	5	0.001	0.025	
chr6	30039403	30039442	5	0.003	0.027	***RNF39***
chr6	160023581	160024002	5	0.003	0.027	
chr6	33091596	33091751	3	0.004	0.030	***HLA-DPB2***
chr6	32525805	32525813	2	0.004	0.030	***HLA-DRB6***
chr6	29895116	29895260	5	0.004	0.030	*HCG4B*
chr6	139012860	139013146	4	0.004	0.030	*FLJ46906*
chr6	29894050	29894195	2	0.005	0.031	*HCG4B*
chr6	110720501	110721349	5	0.006	0.031	*DDO*
chr6	2768054	2770510	3	0.006	0.032	*WRNIP1*
chr6	160182438	160182624	4	0.007	0.033	*LOC100129518*
chr6	29723301	29723407	4	0.007	0.034	
chr6	6894084	6894182	3	0.009	0.039	
chr6	30079203	30079280	4	0.011	0.042	***TRIM31***
chr6	41068646	41068724	4	0.013	0.045	*ADCY10P1*
chr6	29520752	29520774	2	0.014	0.046	
chr6	29854740	29854799	2	0.016	0.048	*HLA-H*
chr15	45028083	45028595	5	0.001	0.0261	***TRIM69***

Replication sample

CHR	Start	End	# CpG	p.value	FDR	Near Gene
chr6	29911550	29913343	3	0.0002	0.0347	***HLA-A***
chr6	30038998	30039524	14	0.0001	0.0172	***RNF39***
chr6	30079203	30079280	3	0.0050	0.0984	***TRIM31***
chr6	32525805	32525813	2	0.0040	0.0928	***HLA-DRB6***
chr6	32526027	32526260	2	0.0157	0.1647	*HLA-DRB6*
chr6	33048254	33048310	4	0.0074	0.1164	*HLA-DPB1*
chr6	33091634	33091751	2	0.0028	0.0842	***HLA-DPB2***
chr15	45028270	45028595	2	0.0460	0.2707	***TRIM69***

Note: Bolded genes are consistent in the discovery and replication sample sets

Supplementary Table 2 Top 748 CpG sites from epigenome-wide association analysis compared IDU+/HCV+ to IDU-/HCV-

probe	CHR	MAPINFO	_REFGENE_N	REFGENE_GROUP	Coefficients	Std_error	Pvalue
cg07839457	16	57023022	*NLRC5*	TSS1500	-0.0763	0.0119	5.76E-10
cg22107533	15	45028083	*RIM69;TRIM6*	1500;TSS1500	-0.0353	0.00552	6.04E-10
cg10439456	15	45028270	*RIM69;TRIM6*	1500;TSS1500	-0.0489	0.00843	1.57E-08
cg22917487	3	39322103	*X3CR1;CX3CR*	S200;5UTR;TSS1500	0.0175	0.00316	6.64E-08
cg05439368	15	45028098	*RIM69;TRIM6*	1500;TSS1500	-0.0337	0.00615	9.09E-08
cg05349042	1	1.47E+08	*BCL9*	TSS200	0.0118	0.00224	2.43E-07
cg04338890	16	57019755			-0.0132	0.00262	7.64E-07
cg16365445	10	33230686	*ITGB1;ITGB1*	5UTR;5UTR	0.0137	0.00278	1.29E-06
cg21577365	15	22367858	*IM2;LOC727*	SS1500;Body	-0.0368	0.00751	1.55E-06
cg05201185	6	30459139	*HLA-E*	Body	-0.0306	0.00645	3.14E-06
cg24119607	5	1.39E+08	*CXXC5*	5UTR	-0.0183	0.00387	3.22E-06
cg06762442	16	66909285			0.0163	0.00344	3.46E-06
cg09624923	10	33244211	*ITGB1;ITGB1*	5UTR;5UTR	0.0187	0.00399	4.36E-06
cg00385925	16	3095538	*MMP25*	TSS1500	-0.00701	0.0015	4.38E-06
cg19896824	11	1.29E+08			-0.016	0.00346	5.32E-06
cg18808777	6	31431503	*HCP5*	3UTR	-0.0302	0.00653	5.58E-06
cg23637607	3	1.14E+08			-0.0202	0.00437	5.60E-06
cg17046484	17	19269624			-0.0103	0.00222	5.66E-06
cg15807655	7	98869778	*MYH16*	TSS1500	0.0131	0.00285	6.78E-06
cg20300175	17	27918316			-0.0199	0.00434	7.00E-06
cg09253508	19	12721610	*90;ZNF791;ZN*	on;TSS200;5UTR	0.00446	0.000979	7.40E-06
cg15194251	11	2239813			-0.0152	0.00335	8.20E-06
cg15022400	15	45028161	*RIM69;TRIM6*	1500;TSS1500	-0.0196	0.00433	8.40E-06
cg08818207	6	32820355	*TAP1*	Body	-0.0358	0.00793	8.83E-06
cg11302624	5	1.77E+08	*RGS14*	Body	0.0129	0.00286	9.85E-06
cg08252585	2	24931616	*A1;NCOA1;NC*	dy;Body;Body	0.0114	0.00254	1.05E-05
cg22909759	14	65185328	*PLEKHG3*	5UTR	-0.0104	0.00234	1.20E-05
cg11351841	8	1.44E+08			0.0229	0.00515	1.22E-05
cg20664636	17	1959596	*HIC1;HIC1*	SS200;5UTR	-0.0169	0.00382	1.34E-05
cg23615741	10	1.01E+08			-0.0439	0.00996	1.47E-05
cg16411857	16	57023191	*NLRC5*	TSS1500	-0.0204	0.00464	1.55E-05
cg09053247	3	44802549	*F15;KIAA114*	SS1500;Body	0.0403	0.0092	1.64E-05
cg26433582	11	68848232	*TPCN2*	Body	0.0233	0.00533	1.65E-05
cg22071610	22	24086732	*ZNF70*	Body	0.00852	0.00195	1.65E-05
cg27464311	17	46692347	*HOXB8*	TSS200	-0.0071	0.00162	1.68E-05
cg13424029	10	1.01E+08			-0.0261	0.00598	1.70E-05
cg08099136	6	32811251	*PSMB8;PSMB*	Body;Body	-0.028	0.00642	1.74E-05
cg01309328	6	32811253	*PSMB8;PSMB*	Body;Body	-0.0245	0.00561	1.74E-05
cg10517312	15	45027253	*RIM69;TRIM6*	1500;TSS1500	-0.0134	0.00309	1.81E-05
cg04165933	16	49833847	*ZNF423*	Body	-0.0132	0.00303	1.91E-05
h.12.41606343	12	43320076			0.00351	0.000809	1.97E-05
cg08516083	14	65210088	*PLEKHG3*	Body	-0.00934	0.00216	1.98E-05
cg22303930	2	2.04E+08	*CYP20A1*	Body	0.00393	0.000909	2.06E-05

cg26312951	21	42797847	*MX1;MX1*	TSS200;5UTR	-0.046	0.0107	2.17E-05
cg10586087	5	1.48E+08	*SH3TC2*	3UTR	-0.0109	0.00252	2.18E-05
cg01138448	15	45028595	*RIM69;TRIM6*	tExon;5UTR;1stExon	-0.0242	0.00563	2.23E-05
cg13491490	3	55646231	*ERC2*	3UTR	0.0217	0.00504	2.24E-05
cg22102703	21	43346284	*C2CD2;C2CD2*	Body;5UTR	-0.0293	0.00682	2.30E-05
cg00066153	11	1.26E+08	*ACRV1;ACRV1*	R;1stExon;1stExon;1stI	0.00641	0.00149	2.38E-05
cg07326074	16	2510388	*C16orf59*	Body	0.00876	0.00204	2.41E-05
cg02020384	12	68704235	*MDM1*	Body	0.0143	0.00333	2.41E-05
cg24486958	1	2.13E+08			0.0146	0.00342	2.62E-05
cg04581327	17	17687239	*RAI1*	5UTR	-0.00587	0.00138	2.77E-05
cg07140459	3	1.42E+08	*XRN1;XRN1*	Body;Body	-0.00976	0.0023	2.93E-05
cg02678476	4	1.84E+08			-0.00695	0.00165	3.15E-05
cg19461344	2	27485922	*C30A3;SLC30*	stExon;5UTR	-0.0102	0.00242	3.25E-05
cg22583148	17	46674041	*C404266;LOC*	Body;Body;Body;Body	-0.0118	0.00281	3.57E-05
cg09154880	6	32813715	*TAP1;PSMB8*	ody;TSS1500	-0.0235	0.00561	3.63E-05
cg22553289	17	33469403	*NLE1;NLE1*	SS200;TSS200	-0.00179	0.000427	3.64E-05
cg21081878	21	38334730	*HLCS*	5UTR	0.0244	0.00588	4.15E-05
cg14259466	10	1.35E+08	*18;ADAM8;AD*	;TSS1500;TSS1500	-0.0151	0.00363	4.20E-05
cg26831566	1	20693014			0.0098	0.00236	4.23E-05
cg26074940	3	66293457	*;SLC25A26;S*	TR;Body;Body	-0.0131	0.00316	4.34E-05
cg04067276	6	1.07E+08	*AIM1*	TSS1500	-0.0061	0.00147	4.41E-05
cg04840203	5	1.56E+08			-0.0188	0.00455	4.63E-05
cg05429117	10	83630419			0.0213	0.00516	4.70E-05
cg24566824	11	1.34E+08	*GLB1L2*	3UTR	-0.00988	0.0024	4.86E-05
cg22339338	1	1.6E+08	*TAGLN2*	5UTR	0.0133	0.00323	5.11E-05
cg18019810	2	2.37E+08	*AGAP1;AGAP1*	Body;Body	0.0146	0.00356	5.20E-05
cg13280359	4	1353105	*KIAA1530*	Body	0.0121	0.00295	5.28E-05
cg06412669	17	78735550	*RPTOR;RPTOR*	Body;Body	0.02	0.0049	5.55E-05
cg08826184	12	44973266	*L2;NELL2;NE*	dy;Body;Body;Body	0.0153	0.00374	5.55E-05
cg16850419	17	74237210	*RNF157*	TSS1500	-0.00706	0.00173	5.70E-05
cg25214187	11	1.34E+08			-0.017	0.00417	5.81E-05
cg08000336	1	36621206	*MAP7D1*	TSS1500	-0.00393	0.000965	5.84E-05
cg12204343	5	87964048	*;LOC645323;*	dy;Body;Body	0.0296	0.00726	5.85E-05
cg26573274	13	1.1E+08			-0.0105	0.00257	5.99E-05
cg16574134	10	1.03E+08	*X2;PAX2;PA*	TR;3UTR;3UTR;3UTR	-0.00741	0.00182	6.00E-05
cg11803771	11	65408566	*SIPA1;SIPA1*	Body;Body	-0.013	0.0032	6.09E-05
cg15009294	1	2.15E+08	*CENPF*	Body	0.0136	0.00335	6.10E-05
cg23326197	7	99382370	*CYP3A4*	TSS1500	-0.0117	0.00288	6.35E-05
cg05386387	18	71815086	*XO15;C18orf*	tExon;TSS1500;5UTR	0.00144	0.000354	6.38E-05
cg13502346	14	65170209	*PLEKHG3*	TSS1500	-0.00836	0.00206	6.40E-05
cg19320261	1	64409138	*ROR1;ROR1*	Body;Body	0.0206	0.0051	6.51E-05
cg15434292	18	77567564			0.0104	0.00257	6.52E-05
cg10919522	14	74227441	*4orf43;C14or*	UTR;TSS1500	-0.0167	0.00413	6.58E-05
cg09021952	1	1.73E+08			0.0121	0.003	6.73E-05
cg20408505	6	29911494	*HLA-A*	Body	-0.0234	0.00579	6.88E-05
cg16316407	1	2.32E+08	*C1;DISC1;TSN*	Body;Body;Body;Body;	0.00853	0.00212	6.99E-05
cg06960794	6	1.49E+08			-0.00683	0.0017	7.14E-05

cg02426178	19	10747142	*C44A2;SLC44*	Body;Body	0.00864	0.00215	7.23E-05
cg25308562	13	64516824			-0.0239	0.00595	7.34E-05
cg27160171	13	45237985			-0.0205	0.00511	7.35E-05
cg23394391	1	8409065			0.00439	0.00109	7.50E-05
cg23086176	1	25226129	*RUNX3;RUNX3*	3UTR;3UTR	-0.00983	0.00245	7.52E-05
cg11270042	17	26219919	*C17orf108*	5UTR	-0.00694	0.00173	7.67E-05
cg11187245	6	31323397	*HLA-B*	Body	-0.0271	0.00677	7.72E-05
cg13237033	14	61116216	*SIX1*	TSS200	0.0092	0.0023	7.81E-05
cg03453880	2	85049005	*C2orf89*	3UTR	0.00831	0.00208	8.29E-05
cg08617135	6	9516073			-0.0117	0.00293	8.38E-05
cg08140412	12	52014778	*SCN8A*	5UTR	-0.0165	0.00415	8.44E-05
cg08159663	16	57022486	*NLRC5*	TSS1500	-0.0235	0.00589	8.54E-05
cg05934682	19	50394298	*IL4I1;IL4I1*	Body;Body	0.0185	0.00465	8.81E-05
cg05916196	1	2.07E+08	*IL19*	1stExon	0.00648	0.00164	9.08E-05
cg22160883	5	1.39E+08	*MATR3;SNHG4*	1500;TSS1500	-0.00983	0.00248	9.12E-05
cg10325659	14	94423950	*ASB2*	TSS200	-0.00963	0.00243	9.13E-05
cg14615914	9	7380374			0.0151	0.00381	9.20E-05
cg06782686	14	52535964	*NID2*	TSS200	-0.00681	0.00172	9.41E-05
cg12394377	1	24514218	*RA;IL28RA;IL2*	0;TSS1500;TSS1500	-0.00571	0.00144	9.45E-05
cg27504460	2	1.88E+08			-0.019	0.00482	9.53E-05
cg04023663	7	7031200			0.0212	0.00536	9.60E-05
cg14570479	19	3199082	*NCLN*	Body	0.00466	0.00118	9.70E-05
cg18112794	21	15057058			-0.0212	0.00538	9.81E-05
cg14958969	2	1942814	*MYT1L*	Body	-0.0111	0.00281	9.85E-05
cg05139434	1	41328180	*CITED4*	TSS200	0.00321	0.000815	9.86E-05
cg11521274	1	1.98E+08	*NEK7*	5UTR	-0.0231	0.00585	9.87E-05
cg18302606	19	39904402	*PLEKHG2*	5UTR	-0.0225	0.00571	1.00E-04
cg18125510	14	1.01E+08	*WARS;WDR2*	5UTR;TSS1500;1stExon	-0.0165	0.0042	0.000103
cg16622651	6	76724431	*IMPG1*	Body	-0.019	0.00483	0.000103
cg00817377	7	73441057	*ELN;ELN;ELN*	0;TSS1500;TSS1500;TSS	-0.0119	0.00302	0.000103
cg08878352	6	30035220	*PPP1R11*	1stExon	0.00603	0.00153	0.000103
cg23924326	4	1.49E+08	*ARHGAP10*	Body	-0.00579	0.00147	0.000104
cg11908294	18	46067903	*A0427;KIAA0*	5UTR;5UTR	-0.0113	0.00288	0.000105
cg00004089	17	74678836	*A7;MXRA7;M*	dy;3UTR;Body	0.0119	0.00303	0.000108
cg12737588	15	70546706			-0.0013	0.000332	0.000114
cg22930808	3	1.22E+08	*3L;PARP9;PA*	500;5UTR;5UTR;5UTR;	-0.0697	0.0178	0.000115
cg03770248	15	80762691	*ARNT2*	Body	-0.00678	0.00174	0.000116
cg14847975	12	26986502	*ITPR2*	TSS1500	-0.003	0.000768	0.000117
cg19059861	20	31823761	*PLUNC;PLUNC*	SS200;TSS200	0.0182	0.00468	0.00012
cg03087163	5	1.79E+08			0.00719	0.00185	0.000122
cg21441076	17	48929520			0.00647	0.00166	0.000123
cg23005227	3	50645426	*CISH;CISH*	Body;Body	0.0205	0.00528	0.000125
cg14593550	12	7053164	*C12orf57*	TSS200	0.0112	0.0029	0.000125
cg01090609	18	29264252	*B4GALT6*	Body	-0.00752	0.00194	0.000127
cg14906976	21	45177538	*PDXK*	3UTR	-0.00642	0.00165	0.000127
cg23505299	5	1.49E+08	*CSF1R*	Body	-0.00819	0.00211	0.000128
cg04861640	6	28234605	*37;ZNF187;ZN*	0;TSS200;TSS200	0.0175	0.00451	0.000128

cg04901763	2	2.32E+08	*CAB39;CAB39*	xon;TSS1500;TSS1500	-0.00368	0.000949	0.000129
cg00550976	2	2.03E+08	*S2CR4;ALS2C*	TSS200;Body	-0.0048	0.00124	0.00013
cg18991321	7	1E+08	*AGFG2*	Body	-0.01	0.00258	0.00013
cg11200656	17	75956447			0.052	0.0134	0.000132
cg04189149	16	32898433			-0.0128	0.00332	0.000134
cg24095374	5	1.77E+08	*RGS14*	3UTR	0.0103	0.00268	0.00014
cg17952824	10	44287023	*HNRNPA3P1*	TSS1500	-0.0148	0.00384	0.000141
cg27000161	16	87688479	*JPH3*	Body	0.0023	0.000599	0.000143
cg16657479	2	1.32E+08	*50786;LOC15*	UTR;1stExon	0.0192	0.00498	0.000143
cg08373187	5	1.35E+08	*CXCL14*	Body	-0.015	0.0039	0.000144
cg03738384	16	84628969	*COTL1*	Body	-0.0276	0.00719	0.000146
cg25103160	12	76206788			-0.0122	0.00316	0.000148
cg20600850	12	6712515	*CHD4*	Body	0.00844	0.0022	0.000152
cg26965547	17	73893447	*TRIM65*	TSS1500	-0.0063	0.00164	0.000154
cg12945444	8	11536969			0.0115	0.003	0.000157
cg21488876	19	5250376	*PTPRS;PTPRS*	;Body;Body;Body	0.0135	0.00353	0.000161
cg27370808	10	8079737			-0.0104	0.00272	0.000161
cg15641364	1	1.6E+08	*TAGLN2*	5UTR	0.0289	0.00756	0.000162
cg26990406	7	178829			-0.00871	0.00228	0.000166
cg00118342	2	1.07E+08	*UXS1*	Body	-0.00844	0.00222	0.000167
cg16650530	1	32538413	*TMEM39B*	TSS200	0.00329	0.000865	0.000167
cg11085282	22	46262203			0.0143	0.00374	0.000168
cg09178700	15	28344507	*OCA2*	TSS200	-0.012	0.00314	0.000168
cg06940299	16	75147500	*LDHD;LDHD*	Body;Body	-0.0067	0.00176	0.000169
cg09216823	3	1.93E+08	*P13A4;ATP13*	UTR;1stExon	0.0233	0.00614	0.00017
cg27505492	12	1.33E+08			0.0501	0.0132	0.00017
cg05137103	6	29396182	*OR11A1*	TSS1500	0.00793	0.00208	0.00017
cg01910478	14	76373678	*TTLL5*	Body	0.00748	0.00197	0.000171
cg07981328	1	21724633			0.0571	0.015	0.000171
cg14195992	8	48265917	*KIAA0146*	Body	-0.0137	0.00361	0.000173
cg14782970	12	53189960	*KRT3*	TSS200	-0.0189	0.00497	0.000174
cg22670675	1	15533433	*EM51;TMEM*	5UTR;5UTR;5UTR	-0.0139	0.00365	0.000175
cg23494957	14	64761548	*SR2;ESR2;ESR*	00;TSS1500;5UTR	-0.00372	0.000979	0.000175
cg09856996	14	1.05E+08	*KIF26A*	Body	-0.107	0.0283	0.000178
cg14319773	19	292360	*2C;PPAP2C;PP*	);TSS1500;TSS1500	-0.0174	0.00459	0.000181
cg21127580	6	901081			0.0222	0.00586	0.000182
cg15655935	11	59815177	*PLAC1L*	3UTR	0.0163	0.0043	0.000184
cg08122652	3	1.22E+08	*3L;PARP9;PA*	500;5UTR;5UTR;5UTR;	-0.0566	0.0149	0.000184
cg16565901	17	78735596	*RPTOR;RPTOR*	Body;Body	0.0201	0.00532	0.000186
cg02845988	12	93836455	*UBE2N*	TSS1500	-0.0281	0.00743	0.000188
cg09304040	12	56360607	*DK2;CDK2;SIL*	n;5UTR;TSS1500;1stExo	0.00306	0.00081	0.000189
cg08756121	8	8820884			-0.0179	0.00474	0.000189
cg12303582	10	3169516	*PFKP*	Body	-0.00668	0.00177	0.00019
cg01894064	4	1.54E+08	*TRIM2*	TSS1500	-0.0123	0.00325	0.000193
cg06790674	8	1.44E+08			0.0139	0.00369	0.000194
cg10196650	21	44588747	*CRYAA*	TSS1500	0.00452	0.0012	0.000194
cg26487031	11	1.18E+08	*MPZL2;MPZL2*	Body;Body	-0.00993	0.00263	0.000195

cg21703322	11	1.11E+08	*LAYN*	TSS200	-0.0125	0.0033	0.000195
cg19459791	15	65363022			0.0161	0.00427	0.000197
cg20380465	5	13919497	*DNAH5*	Body	0.00505	0.00134	0.000197
cg16421612	18	2571989	*'DC80;METTL*	UTR;TSS1500	-0.00329	0.000873	0.000198
cg09251764	17	6659070	*XAF1;XAF1*	;S200;TSS200	-0.0126	0.00334	0.000199
cg14331029	14	96658912			-0.0348	0.00925	0.000201
cg14632586	6	19844462			-0.0151	0.00403	0.000202
cg05249026	16	11031524	*DEXI*	3UTR	-0.0104	0.00276	0.000204
cg16849609	2	1.2E+08	*C1QL2*	TSS200	0.011	0.00292	0.000205
cg17341113	11	69237570			0.0079	0.0021	0.000206
cg25469474	4	2749501	*TNIP2;TNIP2*	Body;Body	-0.0167	0.00446	0.000211
cg14911132	11	61596755	*FADS2*	Body	-0.00743	0.00198	0.000211
cg22121647	7	1.35E+08	*TMEM140*	5UTR	-0.0152	0.00406	0.000211
cg02373380	18	61603746			0.00393	0.00105	0.000213
cg03596645	17	37411353	*LOC90110*	TSS1500	-0.0063	0.00168	0.000213
cg00945108	1	12806027	*C1orf158*	TSS200	0.0209	0.00557	0.000213
cg04292615	19	53290079	*ZNF600*	TSS200	0.0218	0.00581	0.000213
cg04921989	2	1.32E+08			-0.014	0.00375	0.000215
cg11437253	3	1.47E+08			-0.00859	0.00229	0.000215
cg11207763	11	69517471	*FGF19*	Body	0.0149	0.00399	0.000215
cg26604359	19	15586845	*PGLYRP2*	Body	-0.00795	0.00213	0.000216
cg18714086	12	1.12E+08			0.0114	0.00306	0.000219
cg03082924	19	18218106	*MAST3*	Body	0.0151	0.00403	0.000221
cg16495149	7	1.11E+08			-0.0111	0.00297	0.000222
cg19988235	2	9945589			-0.0101	0.00271	0.000223
cg13759676	6	1.29E+08	*'TPRK;PTPRK*	Body;Body	-0.00961	0.00257	0.000223
cg00221794	2	20776620			-0.0199	0.00533	0.000224
cg23163573	2	1.09E+08	*LT1C2;SULT1*	tExon;1stExon;5UTR	0.0287	0.00771	0.000227
cg22354132	1	55273637	*orf177;C1orf1*	Body;Body	0.0122	0.00327	0.000227
cg01082299	6	31431969	*HCP5*	3UTR	-0.0294	0.0079	0.000229
cg00569896	4	204382			0.0345	0.00926	0.000229
cg23637705	17	7307466	*C17orf61*	TSS200	-0.00206	0.000554	0.000232
cg18076767	11	86453358			-0.00976	0.00262	0.000232
cg17304222	5	1.8E+08	*FLT4;FLT4*	;1500;TSS1500	0.00435	0.00117	0.000232
cg11344744	2	97526122	*SEMA4C*	3UTR	-0.0152	0.00408	0.000237
cg23712286	16	75182244	*ZFP1*	TSS200	-0.00417	0.00112	0.000242
cg13810707	7	1.22E+08	*;2;CADPS2;C/*	dy;Body;Body	-0.0163	0.00439	0.000244
cg00074145	15	53075505	*ONECUT1*	Body	-0.0274	0.0074	0.000245
cg09972864	13	22248760	*FGF9*	Body	0.00673	0.00181	0.000246
cg25813630	17	1878100	*RTN4RL1*	Body	0.00695	0.00187	0.000246
cg27319917	16	48592694	*N4BP1*	Body	0.033	0.00889	0.000247
cg24874638	3	1.56E+08	*C3orf33*	TSS200	-0.00388	0.00105	0.000248
cg26708638	19	56652447	*ZNF444*	TSS200	0.00109	0.000293	0.00025
cg26800893	11	67184596	*'PGD1;ATPGL*	5UTR;Body	0.0126	0.00339	0.00025
cg08472795	2	1.29E+08	*HS6ST1*	Body	0.0335	0.00904	0.000252
cg18614381	19	31844182			0.00992	0.00268	0.000253
cg02340056	19	55141979	*B1;LILRB1;LIL*	JTR;5UTR;5UTR;5UTR;:	-0.0113	0.00305	0.000255

cg20196966	6	47445060	*CD2AP*	TSS1500	-0.007	0.00189	0.000256
cg11635407	17	49353246	*UTP18*	Body	0.00807	0.00218	0.000257
cg26980937	14	56991775			-0.0626	0.0169	0.000258
cg22539431	7	1.27E+08	*SND1*	Body	0.0161	0.00434	0.000258
cg22107986	5	73980935	*HEXB*	TSS200	0.00296	0.000802	0.00026
cg15770046	19	35764710	*USF2;USF2*	Body;Body	0.00948	0.00257	0.000261
cg07314829	1	1.12E+08	*ADORA3*	Body	-0.0104	0.00281	0.000264
cg22276571	20	17549599	*DSTN;DSTN*	;1500;TSS1500	-0.0116	0.00314	0.000264
cg08199563	19	55816212	*BRSK1*	Body	0.00661	0.00179	0.000272
cg14791799	13	24227716	*RSF19;TNFRS*	Body;Body	0.0131	0.00356	0.000273
cg14200725	12	49942757	*KCNH3*	Body	-0.00856	0.00233	0.000274
cg13696743	14	57287885	*OTX2OS1*	Body	0.0141	0.00384	0.000276
cg02829285	17	68099642	*16;KCNJ16;KC*	;TSS1500;5UTR	-0.0085	0.00231	0.000281
cg04265672	5	1.79E+08	*RNF130*	Body	-0.00942	0.00257	0.000283
cg04216235	5	1.73E+08			0.0115	0.00312	0.000283
cg15696662	1	46669221	*rf190;POMGI*	stExon;Body	-0.00664	0.00181	0.000285
cg16148454	16	69385606	*TMED6*	1stExon	0.00638	0.00174	0.000286
cg13717333	12	1.22E+08	*BCL7A;BCL7A*	5UTR;5UTR;1stExon	0.00287	0.000783	0.000287
cg25806932	2	4051987			-0.0188	0.00512	0.000289
cg01566028	9	1.23E+08	*RAP2;CDK5R*	Body;Body	0.00632	0.00172	0.00029
cg20373634	10	1.04E+08	*NOLC1*	TSS1500	-0.0123	0.00336	0.00029
cg01734786	4	1.63E+08	*TL5;FSTL5;FST*	;TSS1500;TSS1500	-0.0067	0.00183	0.00029
cg12921795	14	1.03E+08	*90AA1;HSP90*	stExon;5UTR;TSS1500;	-0.00473	0.00129	0.000291
cg03855388	11	68580018	*CPT1A;CPT1A*	Body;Body	0.00231	0.000631	0.000291
cg13733008	4	78088735	*CCNG2*	3UTR	0.00876	0.00239	0.000292
cg10093103	14	1.02E+08			0.00873	0.00238	0.000294
cg24694018	1	1.45E+08	*POLR3GL*	Body	-0.0233	0.00637	0.000295
cg23664192	6	34725288	*PC;SNRPC;SN*	R;Body;1stExon	0.00207	0.000565	0.000295
cg22014887	4	2749539	*TNIP2;TNIP2*	Body;Body	-0.0141	0.00384	0.000296
cg14755852	8	23386014	*SLC25A37*	TSS1500	-0.00914	0.00249	0.000297
cg21140633	17	15600659			-0.00743	0.00203	0.000302
cg22591006	15	97814549			0.0081	0.00222	0.000304
cg26711333	8	8821019			-0.0127	0.00347	0.000305
cg13335567	8	71127379	*NCOA2*	Body	-0.0203	0.00557	0.000305
cg04784948	2	24148492	*ATAD2B*	Body	-0.00947	0.0026	0.000308
cg11004890	20	3218500	*SLC4A11*	TSS200	0.0232	0.00635	0.000308
cg09657185	6	34003954	*GRM4*	Body	0.012	0.00329	0.000309
cg14980667	5	1.73E+08			-0.00942	0.00258	0.00031
cg19633390	13	1.1E+08			0.0151	0.00415	0.000312
cg08973950	7	1083309	*50;C7orf50;C*	dy;Body;Body	-0.0237	0.00651	0.000314
cg24654228	7	1.39E+08	*HAV1;ZC3HA*	Body;Body	0.0128	0.00353	0.000315
cg08638044	15	27819924			0.015	0.00412	0.000315
cg16108059	14	81421989	*;TSHR;TSHR;*	xon;1stExon;5UTR;1st	-0.0159	0.00438	0.000316
cg17050097	17	60783168	*CH10;MARC*	Body;Body	-0.00702	0.00193	0.000318
cg02171008	11	8703884	*RPL27A*	TSS200	-0.00284	0.00078	0.000319
cg11511908	11	61919362	*ICENP;INCEN*	Body;Body	0.00531	0.00146	0.00032
cg12308965	11	67291387	*CABP2;CABP2*	1500;TSS1500	-0.00802	0.0022	0.00032

cg18530075	2	25265699	*EFR3B*	Body	0.00507	0.0014	0.000321
cg27453365	11	1.08E+08	*ATM;NPAT*	SS1500;Body	0.00712	0.00196	0.000322
cg04724540	1	1.67E+08	*ILDR2*	Body	-0.0072	0.00198	0.000323
cg18223379	20	31619647	*BPIL3*	Body	0.00984	0.00271	0.000324
cg17901584	1	55353706	*DHCR24*	TSS1500	0.0268	0.00738	0.000328
cg14662970	1	1.09E+08	*SLC25A24*	Body	-0.00212	0.000583	0.000328
cg02897042	10	95720710	*PIPSL*	Body	0.00941	0.00259	0.000329
cg25556432	2	2.4E+08			0.0127	0.0035	0.000333
cg27614534	18	56296496	*ALPK2*	TSS1500	-0.00761	0.0021	0.000334
cg23058037	7	1.59E+08	*VIPR2*	Body	-0.0322	0.00887	0.000334
cg19329389	15	64680051	*TRIP4;TRIP4*	UTR;1stExon	-0.00404	0.00112	0.000335
cg15024566	2	55496144	*F2;MTIF2;M*	xon;5UTR;5UTR	0.00331	0.000913	0.000335
ch.5.3304132F	5	1.73E+08	*STC2*	Body	0.00325	0.000897	0.000335
cg26064870	8	1.03E+08	*LD;NCALD;NC*	TR;5UTR;5UTR;5UTR;5	-0.013	0.00358	0.000336
cg18960469	10	1.02E+08			0.00649	0.00179	0.000337
cg17626301	6	32820201	*TAP1*	Body	-0.0183	0.00504	0.000339
cg20759345	11	86745909			0.0114	0.00315	0.00034
cg27032101	11	312841	*IFITM1*	TSS1500	-0.0284	0.00785	0.00034
cg25939853	12	1.33E+08			-0.0224	0.00617	0.00034
cg00583291	5	1.8E+08			-0.00743	0.00205	0.000342
cg13388983	11	66824140	*RHOD*	TSS200	-0.00735	0.00203	0.000342
cg18234224	1	1.73E+08			0.00974	0.00269	0.000343
cg09432376	22	36044226	*APOL6*	TSS200	-0.0155	0.00429	0.000345
cg14930335	2	2.37E+08	*GAP1;AGAP1*	Body;Body	0.0195	0.00539	0.000345
cg16068812	21	44528396	*F1;U2AF1;U2*	);TSS1500;TSS1500	-0.0247	0.00683	0.000345
cg05687498	5	1.4E+08			0.0405	0.0112	0.000346
cg12821980	11	74702244	*NEU3*	Body	-0.0105	0.00289	0.000349
cg24969820	3	98497864	*ST3GAL6*	Body	-0.00762	0.00211	0.000353
cg09092093	5	58335954	*4D;PDE4D;PD*	dy;Body;Body	-0.006	0.00166	0.000355
cg25966198	14	20904303	*KLHL33*	TSS1500	0.00652	0.00181	0.000356
cg13469633	4	1.86E+08	*KIAA1430*	TSS1500	-0.0052	0.00144	0.000356
cg00956639	1	35659678	*SFPQ*	TSS1500	-0.00863	0.00239	0.000357
cg25992382	14	93066811	*RIN3*	Body	0.0109	0.00302	0.00036
cg14552404	15	25321097	*16-11;SNORI*	ody;TSS1500	-0.0212	0.00588	0.000361
cg00826921	3	1.44E+08	*3orf58;C3orf5*	Body;TSS200	0.00409	0.00114	0.000361
cg19076265	8	25090321	*DOCK5*	Body	-0.0108	0.00301	0.000362
cg05957730	2	71361235	*MPHOSPH10*	Body	0.0152	0.00421	0.000363
cg27128489	16	46865867	*C16orf87*	TSS1500	0.0294	0.00816	0.000364
cg21558893	15	75950332	*SNX33*	3UTR	-0.00509	0.00141	0.000365
cg05695311	8	37887949	*EIF4EBP1*	TSS200	0.00281	0.00078	0.000367
cg26855689	17	79668668	*HGS*	3UTR	0.00342	0.000949	0.000369
cg09717585	16	87517852	*ZCCHC14*	Body	-0.0167	0.00464	0.00037
cg17101703	2	69793701	*AAK1*	Body	0.0117	0.00324	0.000372
cg18295183	5	1.61E+08	*BRG2;GABR*	;5UTR;5UTR;5UTR;1st	-0.0124	0.00343	0.000373
cg00622149	14	36295585	*BRMS1L*	TSS200	0.00456	0.00127	0.000375
cg05002642	20	23969429	*GGTLC1*	TSS200	0.00429	0.00119	0.000375
cg16815330	1	1.52E+08	*SNX27*	Body	-0.0122	0.00338	0.000378

cg18752854	2	2.19E+08	*TNS1*	TSS1500	0.0122	0.00338	0.000378
cg27662789	12	43666281			0.0485	0.0135	0.000381
cg04490037	7	50633773	*DDC*	TSS1500	0.0171	0.00476	0.000383
cg27130075	13	1.11E+08	*COL4A1*	Body	-0.00968	0.0027	0.000387
cg22179059	6	29714945	*85830;LOC28*	Body;Body	-0.0132	0.00369	0.000388
cg19115490	6	37483925			0.00664	0.00185	0.00039
cg06745004	11	62521749	*ZBTB3*	TSS200	-0.00277	0.000772	0.00039
cg11017057	3	51706056	*64;TEX264;TE*	TR;5UTR;Body	0.00371	0.00103	0.000391
cg03950476	1	53019814	*1;ZCCHC11;Z*	0;TSS1500;TSS1500	-0.0183	0.00511	0.000391
cg18550509	15	1E+08	*LYSMD4*	5UTR	-0.00488	0.00136	0.000394
cg24554987	7	1.56E+08			0.0283	0.00791	0.000395
cg04663692	12	97885669	*MST;MIR125*	ody;TSS200	0.0118	0.00329	0.000396
cg04768449	12	94495274			0.0037	0.00103	0.000396
cg11330307	15	92628595	*CO3A1;SLCO3*	Body;Body	-0.00876	0.00245	0.000399
cg17184479	10	94790819	*EXOC6;EXOC6*	Body;Body	-0.0152	0.00425	0.000401
cg22652999	4	3897762			0.00743	0.00208	0.000402
cg17446339	11	20385254	*2;HTATIP2;H*	500;1stExon;5UTR;5UT	-0.0759	0.0212	0.000402
cg14854901	5	72793969	*BTF3;BTF3*	1500;TSS1500	-0.00634	0.00177	0.000403
cg16749570	1	17026817	*ESPNP*	Body	0.00705	0.00197	0.000403
cg09853936	1	3388196	*ARHGEF16*	Body	0.0197	0.00552	0.000403
cg02194398	4	1.43E+08	*IL15;IL15*	1500;TSS1500	0.0135	0.00378	0.000404
cg20102336	12	1.13E+08	*OAS3*	Body	-0.00436	0.00122	0.000404
cg17782848	5	1.7E+08	*P1;KCNIP1;KC*	dy;Body;Body	0.00755	0.00211	0.000404
cg05105745	13	1.13E+08	*TP11A;ATP11*	Body;Body	0.0105	0.00295	0.000406
cg07170300	12	59434386			0.014	0.00393	0.000407
cg03608515	13	98112404	*RAP2A*	Body	0.0125	0.00349	0.000407
cg18581191	8	1.46E+08	*KIAA1688*	Body	-0.00278	0.000779	0.000407
cg19768727	2	2.43E+08	*ATG4B;ATG4E*	Body;Body	-0.00563	0.00158	0.000408
cg03046819	1	2.02E+08	*HISA4;SHISA4*	Body;Body	-0.0118	0.00331	0.000409
cg16913841	10	22725451			0.033	0.00925	0.000409
cg19381766	7	5597647			-0.0129	0.0036	0.000414
cg16006965	3	1.12E+08	*GCET2;GCET2*	1500;TSS1500	-0.0268	0.0075	0.000415
cg02612963	9	1.14E+08	*NF483;ZNF48*	S200;TSS200	-0.00771	0.00216	0.000416
cg11420667	11	1750353	*HCCA2*	Body	-0.0201	0.00565	0.000422
cg14055655	19	50117794	*PRR12*	Body	0.0159	0.00447	0.000426
cg16523868	3	1.41E+08	*ACPL2;ACPL2*	5UTR;5UTR	0.0153	0.00429	0.000429
cg07309864	17	73238441	*GGA3;GGA3*	Body;Body	-0.0154	0.00432	0.00043
cg08229366	7	1.3E+08	*1;MEST;MES*	1500;5UTR;TSS1500	0.0163	0.00458	0.000431
cg04131610	3	46411447	*CCR5;CCR5*	S200;TSS200	0.0112	0.00315	0.000432
cg07360182	1	1.8E+08	*QSOX1;QSOX1*	S200;TSS200	-0.00545	0.00153	0.000433
cg17111087	1	1.67E+08	*CD247;CD247*	Body;Body	0.0184	0.00518	0.000434
cg12809098	8	1.42E+08	*DENND3*	Body	0.0115	0.00323	0.000436
cg15002250	2	2.37E+08	*AGAP1;AGAP1*	Body;Body	0.0121	0.00341	0.000439
cg01234420	22	46453808	*LOC150381*	Body	-0.0184	0.00518	0.00044
cg06782692	10	1.34E+08	*TK32C;STK32*	UTR;1stExon	0.00226	0.000637	0.00044
cg16517579	16	67598143	*CTCF*	5UTR	0.0164	0.00462	0.000443
cg20214319	7	65670151	*TPST1*	TSS200	0.00225	0.000633	0.000447

cg07392324	7	1.08E+08			-0.0264	0.00746	0.000448
cg24070837	19	37108188	*ZNF382*	Body	0.00783	0.00221	0.00045
:h.13.80601193I	13	81703192			-0.00705	0.00199	0.000451
cg17521020	6	29617613			0.00329	0.000928	0.000451
cg18065361	8	67344588	*ADHFE1*	TSS200	0.00218	0.000616	0.000456
cg25880537	1	2365337			-0.0155	0.00437	0.000458
cg07552707	20	44519093	*CTSA;NEURL2*	S1500;1stExon;TSS150(	-0.00795	0.00225	0.000459
cg25506106	19	17970744	*\P3;RPL18A;F*	y;1stExon;5UTR	0.00233	0.000659	0.000459
cg08921185	16	2285536	*NASE1L2;E4F*	SS1500;Body	-0.00229	0.000646	0.000459
cg12806892	12	1.13E+08			-0.0105	0.00297	0.000459
cg03598754	17	26711803	*SARM1*	Body	-0.00921	0.0026	0.000461
cg18139174	14	24605759	*PSME1;PSME*	Body;Body	0.0138	0.0039	0.000463
cg12627299	11	66444235	*RBM4B*	Body	-0.00746	0.00211	0.000465
cg15343224	11	1.34E+08			-0.00938	0.00265	0.000466
cg09390792	11	44881444	*TSPAN18*	5UTR	-0.00735	0.00208	0.000474
cg14076258	11	77183807	*PAK1;PAK1*	5UTR;5UTR	-0.0229	0.0065	0.000474
cg05597554	13	46038994	*COG3*	TSS200	-0.00173	0.000489	0.000474
cg19051861	22	50243786			-0.0206	0.00583	0.000476
cg12063688	14	65173327	*PLEKHG3*	5UTR	-0.0102	0.00288	0.000477
cg09985344	16	84648441	*COTL1*	Body	-0.013	0.00367	0.000478
cg06077876	11	64795361	*SNX15;SNX15*	Body;Body	0.00376	0.00106	0.000479
cg15324424	11	62105554	*SRGL1;ASRGL*	Body;Body	-0.00522	0.00148	0.000479
cg27304519	10	14551091			-0.0083	0.00235	0.000479
ch.9.25423502R	9	25433502			-0.0088	0.00249	0.00048
cg16972309	12	1.09E+08	*SSH1;SSH1*	51500;TSS1500	-0.00458	0.0013	0.000482
cg06777516	5	529258			0.0231	0.00656	0.000483
cg01696605	7	1.51E+08			0.0345	0.00979	0.000483
cg09900489	11	1.27E+08	*RREL3;KIRREL*	Body;Body	-0.0109	0.00309	0.000486
cg21093033	8	1.41E+08	*APPC9;TRAPF*	S1500;TSS200	-0.0168	0.00475	0.000486
cg07405330	3	39547215	*MOBP;MOBP*	Body;Body	-0.00928	0.00263	0.000488
cg24693368	2	1.56E+08	*KCNJ3*	TSS1500	0.00748	0.00212	0.000489
cg07084019	2	1.71E+08	*3B;MYO3B;M*	dy;Body;Body	-0.0095	0.0027	0.00049
cg00363312	7	1.39E+08	*HIPK2;HIPK2*	Body;Body	-0.0132	0.00375	0.000491
cg14440600	19	50193021	*T1C;C19orf76*	SS1500;1stExon;5UTR	0.0187	0.0053	0.000492
cg14716311	13	52601031	*TP14C;ALG1*	5UTR;Body	0.00661	0.00188	0.000494
cg01448544	12	1.31E+08	*RIMBP2*	Body	-0.00614	0.00174	0.000496
cg18865990	13	45970273	*SLC25A30*	Body	0.0103	0.00293	0.000496
cg27542552	19	11689488	*CP5;ACP5;ACI*	TR;5UTR;5UTR;1stExor	-0.00687	0.00195	0.000496
cg03753191	13	43566902	*EPSTI1;EPSTI1*	51500;TSS1500	-0.013	0.0037	0.000498
cg20312390	1	2.04E+08	*TP2B4;ATP2B*	5UTR;5UTR	0.0128	0.00365	0.000501
cg19145886	12	1.33E+08	*EP400*	Body	0.00413	0.00118	0.000503
cg15820955	12	48192939	*HDAC7;HDAC*	Body;Body	0.00868	0.00247	0.000505
cg03599575	15	90893182	*GABARAPL3*	TSS1500	0.00866	0.00246	0.000505
cg27446442	1	36546898			0.00909	0.00259	0.000509
cg26477793	10	1.04E+08	*ACTR1A*	Body	0.00279	0.000794	0.000511
cg03946955	1	1.56E+08	*NA;LMNA;LM*	dy;Body;Body	0.00872	0.00248	0.000515
cg23693200	20	4701448	*PRND*	TSS1500	0.00502	0.00143	0.000518

cg05130405	16	29298242			-0.0118	0.00336	0.000519
cg10003974	7	47552912	*TNS3*	5UTR	-0.0139	0.00397	0.00052
cg20739904	5	6629638	*NSUN2*	Body	0.0085	0.00242	0.00052
cg10371643	13	1.1E+08	*MYO16*	Body	0.0112	0.0032	0.000522
cg24321971	11	27406919	*LGR4*	Body	0.0103	0.00293	0.000522
cg27285197	13	1.12E+08			0.00945	0.0027	0.000527
cg20006729	12	7033146	*ATN1*	TSS1500	0.0119	0.00339	0.000528
cg26366107	20	36531652	*STM2L;VSTM2*	stExon;5UTR	0.00709	0.00203	0.00053
cg08182501	2	1.77E+08			-0.0226	0.00647	0.00053
cg12266841	11	45687263	*CHST1*	TSS200	0.00167	0.000478	0.000532
cg15752436	17	20937757	*USP22*	Body	0.00474	0.00136	0.000533
cg23229352	6	1.54E+08			0.0145	0.00415	0.000534
cg18107960	5	76787392	*WDR41*	Body	-0.0184	0.00526	0.000539
cg17608381	6	29911550	*HLA-A*	Body	-0.0547	0.0157	0.000542
cg09296212	9	1.31E+08	*NAIF1*	1stExon	0.00748	0.00214	0.000542
cg21625002	11	22022885			0.00731	0.00209	0.000545
cg16827257	11	2884460			0.00654	0.00187	0.000545
cg16755500	17	27918338			-0.0187	0.00536	0.000545
cg03530983	6	32781171	*HLA-DOB*	Body	0.00826	0.00237	0.000546
cg15538009	14	51304083			-0.0129	0.00369	0.000548
cg20823137	7	56097042	*PSPH*	5UTR	-0.0102	0.00291	0.000548
cg25937790	3	1.19E+08	*CDGAP*	Body	-0.0124	0.00355	0.000548
cg12585966	19	17650009	*M129C;FAM12*	Body;Body	-0.0153	0.00438	0.000549
cg23954629	11	1.21E+08	*TBCEL;TBCEL*	5UTR;5UTR	-0.00666	0.00191	0.000552
cg10471876	10	1.13E+08	*C2;NCRNA00*	S200;Body;TSS1500;Bo	0.00379	0.00109	0.000553
cg13167431	22	26988419			-0.0108	0.0031	0.000554
cg25843003	6	31431312	*HCP5*	3UTR	-0.0203	0.00582	0.000555
cg04389164	16	4377470			-0.0093	0.00266	0.000555
cg03627300	2	2.19E+08			0.00731	0.0021	0.000557
cg10580269	2	69056515			0.0107	0.00308	0.000558
cg12532141	12	1.33E+08	*GOLGA3*	Body	0.00636	0.00182	0.00056
cg07111713	13	19171607			-0.0065	0.00186	0.000562
cg20207890	1	38470622	*FHL3*	5UTR	0.00718	0.00206	0.000564
cg14435997	2	1808183	*MYT1L*	Body	0.0104	0.00298	0.000567
cg00333622	20	33459477	*GGT7*	Body	0.00524	0.00151	0.000572
cg07258474	2	11809858	*NTSR2*	1stExon	0.0172	0.00494	0.000579
cg07483650	1	2.49E+08	*OR2T4*	1stExon	0.0118	0.0034	0.00058
cg10057264	4	75174204	*EPGN;EPGN*	stExon;5UTR	0.0145	0.00417	0.000582
cg26636244	7	1.02E+08	*POLR2J*	TSS1500	-0.0111	0.00318	0.000583
cg07638500	3	1.23E+08	*;MYLK;MYLK;*	;Body;Body;Body	-0.0097	0.00279	0.000584
cg18319689	1	46016267	*KR1A1;AKR1A*	1500;TSS1500	-0.00265	0.000763	0.000585
cg24898914	6	32810706	*PSMB8;PSMB8*	Body;Body	-0.013	0.00373	0.000585
cg01127300	22	38614796			-0.0245	0.00706	0.000586
cg09322555	6	32826281	*PSMB9;PSMB9*	Body;Body	-0.0112	0.00321	0.00059
cg07512236	6	1.69E+08			-0.00255	0.000733	0.00059
cg23646343	7	1.39E+08	*HIPK2;HIPK2*	Body;Body	-0.00996	0.00287	0.000592
cg27309098	2	2.37E+08	*AGAP1;AGAP1*	Body;Body	0.0158	0.00454	0.000592

cg01362383	19	47760943	*CCDC9*	5UTR	-0.0169	0.00487	0.000593
cg11850508	17	78172311	*\RD14;CARD1*	Body;Body	-0.00721	0.00208	0.000593
cg03359362	19	47289611	*\5;SLC1A5;SL*	.500;Body;5UTR	-0.00651	0.00188	0.000593
cg03962451	2	1.92E+08	*GLS*	Body	-0.0173	0.005	0.000594
cg14009947	10	12238299	*DC123;NUDT*	tExon;TSS200	-0.00334	0.000964	0.000595
cg20036996	8	1.45E+08	*60;PUF60;PU*	dy;Body;5UTR	-0.00655	0.00189	0.000596
cg23030040	4	3414302	*12;RGS12;RG*	dy;Body;Body	0.00457	0.00132	0.000598
cg25525778	2	1.13E+08	*TMEM87B*	TSS1500	0.0294	0.00848	6.00E-04
cg03130891	1	91550			0.0196	0.00565	0.000601
cg21286701	6	1.28E+08	*C6orf174*	Body	0.014	0.00405	0.000601
cg00966098	16	30596551	*ZNF785*	Body	-0.0143	0.00413	0.000602
cg19314834	18	72201018	*CNDP1*	TSS1500	-0.00612	0.00176	0.000603
cg06604690	12	1.23E+08	*52;CCDC62;C(*	0;TSS200;TSS200	-0.00429	0.00124	0.000605
cg16623710	22	45073049	*;PRR5;PRR5;I*	5UTR;5UTR;5UTR;1stE:	-0.00793	0.00229	0.000605
cg03935116	12	31476565	*M60A;FLJ132*	UTR;TSS1500;5UTR	-0.00962	0.00278	0.000606
cg10848140	21	46268755			0.0193	0.00557	0.000608
cg22160784	2	73460416	*T7;CCT7;C2or*	0;TSS1500;TSS200;TSS	-0.00238	0.000687	0.000608
cg05634649	4	7056300	*TADA2B*	Body	0.00768	0.00222	0.00061
cg11574975	1	1.12E+08	*ADORA3*	Body	-0.016	0.00461	0.00061
cg15552370	5	1.72E+08	*NEURL1B*	3UTR	0.0111	0.00321	0.000614
cg03303515	2	47213998	*TTC7A*	Body	-0.00795	0.0023	0.000618
cg15657641	11	47939769			0.00918	0.00265	0.00062
cg02757488	19	41193662	*NUMBL*	Body	0.0118	0.0034	0.000621
cg19540797	2	3607301	*RNASEH1*	TSS1500	-0.00542	0.00157	0.000624
cg02240030	7	2089880	*1;MAD1L1;M*	dy;Body;Body	0.0105	0.00305	0.000625
cg12045511	15	42193157	*EHD4*	Body	-0.00357	0.00103	0.000627
cg22711792	1	50883329	*DMRTA2*	3UTR	0.00703	0.00204	0.000628
cg17375177	6	29627845	*OG;MOG;MC*	dy;Body;Body;Body;Bo	0.00871	0.00252	0.000628
cg20023898	16	67048469			-0.0128	0.0037	0.00063
cg23241201	19	45580017	*ZNF296*	TSS1500	-0.0105	0.00303	0.000631
cg25935014	12	1.04E+08	*?orf42;C12or*	5UTR;5UTR	-0.0106	0.00308	0.000631
cg10499651	3	12465415	*PPARG;PPARG*	Body;Body;Body	0.0238	0.00688	0.000633
cg18067793	3	1.29E+08			-0.0111	0.0032	0.000635
cg00585776	16	30790892	*ZNF629*	3UTR	0.00816	0.00237	0.000636
cg23680451	10	3823790	*5;KLF6;KLF6;K*	Body;Body;Body	-0.0114	0.0033	0.000637
cg16876219	3	1.97E+08			0.00916	0.00265	0.000637
cg21959757	5	1.15E+08			0.0136	0.00394	0.000637
cg00745735	10	69868860	*MYPN*	TSS1500	-0.0112	0.00325	0.00064
cg15179454	19	2274042			-0.0111	0.00323	0.000641
cg11005651	6	32065890	*TNXB*	Body	0.0103	0.00299	0.000642
cg23479065	3	3843811	*LRRN1*	5UTR	-0.00814	0.00236	0.000651
cg18895336	10	50290996	*C10orf72*	Body	-0.00934	0.00271	0.000652
cg27284331	7	1.06E+08			-0.0354	0.0103	0.000655
cg15892650	5	1.51E+08	*\NXA6;ANXA6*	Body;Body	-0.0102	0.00295	0.000658
cg03549856	1	33593141			0.00411	0.0012	0.000659
cg00676801	2	1.92E+08	*STAT1;STAT1*	5UTR;5UTR	-0.0201	0.00583	0.00066
cg19054184	6	30654762	*9;KIAA1949;K*	R;5UTR;1stExon	-0.00327	0.00095	0.000661

cg05931265	11	1.23E+08	*UBASH3B*	Body	-0.00957	0.00278	0.000662
cg03187444	22	33426343	*SYN3*	5UTR	-0.0155	0.00451	0.000667
cg04197935	17	42631005			-0.00901	0.00262	0.000669
cg08498247	11	14382755			0.00598	0.00174	0.000671
cg27315556	3	1.73E+08	*NLGN1*	TSS1500	-0.00834	0.00243	0.000675
cg03330490	10	94452236	*HHEX*	Body	-0.0159	0.00463	0.000676
cg22629111	1	1.66E+08	*FAM78B*	Body	-0.0136	0.00396	0.000677
cg05757757	10	80008637			-0.0154	0.00448	0.000678
cg10542562	11	1.29E+08			-0.011	0.00321	0.000681
cg10546626	20	57424521	*GNASAS;GNAS*	Body;3UTR	0.01	0.00293	0.000683
cg26689097	2	88355367	*KRCC1*	TSS200	-0.00297	0.000866	0.000686
cg26326298	15	42867856	*TARD9;STARD*	stExon;5UTR	-0.00481	0.0014	0.000687
cg20741134	1	1.81E+08			0.0026	0.000758	0.000688
cg25499099	17	7284662	*TNK1*	5UTR	-0.0103	0.003	0.000689
cg13852948	9	1.38E+08	*COL5A1*	Body	0.0101	0.00295	0.000692
cg24116687	6	1.07E+08	*PRDM1*	Body	0.0145	0.00423	0.000692
cg22975853	10	1.28E+08	*AM12;ADAM*	Body;Body	-0.00598	0.00174	0.000695
cg09666237	17	18128581	*LLGL1*	TSS1500	-0.00393	0.00115	0.000697
cg17723958	12	1.24E+08	*CCDC92*	5UTR	0.021	0.00614	0.000698
cg09757109	11	1.12E+08	*IXDC1;DIXDC*	Body;Body	-0.0158	0.00462	0.000699
cg06734985	11	26849501			-0.0178	0.00519	0.000699
cg03796580	17	80333624			0.02	0.00585	0.000699
cg23514324	3	12329213	*RG;PPARG;PP*	00;TSS200;TSS200	0.00518	0.00151	0.000704
cg15472170	1	12012720	*PLOD1*	Body	0.0145	0.00425	0.000709
cg17262810	10	35676874	*CCNY;CCNY*	Body;5UTR	0.0145	0.00425	0.000713
cg22822599	8	61779045	*CHD7*	3UTR	-0.00807	0.00236	0.000713
cg20145426	1	30179925			0.00762	0.00223	0.000719
cg00611548	6	1.17E+08	*TSPYL1;DSE*	SS1500;5UTR	-0.0196	0.00573	0.000723
cg22809682	17	63549924	*AXIN2*	Body	-0.0632	0.0185	0.000724
cg19576738	4	1.24E+08	*FGF2*	Body	-0.0167	0.0049	0.000725
cg01791034	15	42328501			0.0121	0.00355	0.000726
cg20673481	2	18059454	*KCNS3*	TSS1500	0.005	0.00147	0.000728
cg03775246	5	1.11E+08	*5orf13;C5orf1*	Body;Body;TSS1500	-0.0114	0.00333	0.000728
cg25978487	6	32157150	*PBX2*	Body	0.00659	0.00193	0.00073
cg12901283	17	53045974	*COX11;COX11*	dy;1stExon;1stExon;TS	0.00368	0.00108	0.00073
cg04696274	20	43977531	*SDC4*	TSS1500	-0.00299	0.000877	0.000731
cg03468945	10	1.26E+08	*OAT*	TSS200	-0.00499	0.00146	0.000732
cg22310628	6	29692995	*A-F;HLA-F;HL*	dy;Body;Body	-0.0112	0.00329	0.000733
cg07216529	10	90712739	*ACTA2;ACTA2*	SS200;5UTR	-0.00456	0.00134	0.000737
cg21168914	9	35848961	*EM8B;TMEM*	Body;Body	-0.00484	0.00142	0.000737
cg15076481	7	73664568	*RFC2;RFC2*	Body;Body	-0.00641	0.00188	0.000738
cg14667769	10	1.35E+08			-0.0133	0.00389	0.000739
cg16603064	15	69829848			-0.00629	0.00185	0.000741
cg00999988	2	1.67E+08	*GALNT3*	5UTR	0.0104	0.00304	0.000742
cg21284400	1	1.5E+08	*OTUD7B*	5UTR	-0.0135	0.00397	0.000743
cg09511815	13	73211779			-0.0159	0.00467	0.000746
cg11229273	3	57015101	*ARHGEF3*	Body	0.0123	0.0036	0.000748

cg03834031	22	46465717			-0.011	0.00323	0.000749
cg20405893	10	50822158	*IAT;CHAT;CH/*	TR;5UTR;1stExon;5UTR	0.013	0.00383	0.000749
cg13361798	22	41460015			-0.00671	0.00197	0.000751
cg06363968	16	84629008	*COTL1*	Body	-0.0231	0.00678	0.000751
cg00262280	7	1.38E+08	*CREB3L2*	TSS1500	0.00418	0.00123	0.000751
cg11022791	12	68051679	*DYRK2;DYRK2*	Body;Body	-0.00545	0.0016	0.000752
cg10430444	15	76137847	*3E2Q2;UBE2C*	Body;Body	0.00919	0.0027	0.000753
cg18565717	12	1.33E+08			0.0278	0.00817	0.000754
cg11377484	7	1.29E+08	*5;IRF5;IRF5;II*	0;5UTR;5UTR;5UTR;5U	-0.00684	0.00201	0.000755
cg07098752	10	1.2E+08	*PRLHR*	5UTR	0.0265	0.00778	0.000756
cg12558966	4	4866946			0.00744	0.00219	0.000757
cg05260466	3	27756661			-0.0114	0.00336	0.00076
cg03432151	15	89745000	*\BHD2;ABHD:*	3UTR;3UTR	0.0103	0.00303	0.000762
cg13039585	5	1162588			0.0128	0.00378	0.000764
cg18517055	17	80581701	*WDR45L*	Body	0.0188	0.00554	0.000764
cg17582444	3	1.79E+08	*MFN1*	3UTR	-0.0106	0.00312	0.000767
cg03606176	5	1.18E+08	*DMXL1*	TSS1500	0.00267	0.000787	0.000768
cg13474083	9	1.36E+08			0.00992	0.00292	0.000771
cg04396462	3	63082613			-0.00691	0.00203	0.000771
cg16442226	22	29702854	*L1;GAS2L1;GA*	0;TSS200;TSS200	0.00384	0.00113	0.000771
cg03994820	3	350682	*CHL1*	5UTR	-0.031	0.00914	0.000773
cg27020216	3	1.54E+08	*SGEF*	Body	-0.00828	0.00244	0.000774
cg07083806	2	16083276	*IYCN;MYCNO*	ody;TSS1500	-0.00847	0.0025	0.000774
cg04813697	10	22920025	*PIP4K2A*	Body	-0.0215	0.00634	0.000774
cg26279025	19	55882154	*IL11*	TSS1500	0.0224	0.0066	0.000775
cg06035456	10	71560212	*L13A1;COL13*	1500;TSS1500;TSS150	0.0173	0.00508	0.000776
cg13548265	11	67889607	*CHKA;CHKA*	1500;TSS1500	-0.00682	0.00201	0.000777
cg25383568	19	39217721	*ACTN4*	Body	-0.0142	0.00418	0.000777
cg13846358	9	1.35E+08	*NTNG2*	Body	-0.00767	0.00226	0.000778
cg07323350	11	1.24E+08	*5A;VWA5A;V*	0;TSS200;TSS200	-0.0048	0.00142	0.000779
cg10985970	11	1.3E+08			0.00889	0.00262	0.000781
cg11382394	1	2564504	*MMEL1*	TSS200	0.0108	0.00319	0.000781
cg10149976	14	52535953	*NID2*	TSS200	-0.00668	0.00197	0.000782
cg14213992	19	40503358	*ZNF546*	5UTR	-0.00577	0.0017	0.000783
cg08707078	7	23720292	*16;C7orf46;C7*	dy;Body;Body	-0.00956	0.00282	0.000784
cg04118190	2	96812528	*DUSP2*	TSS1500	-0.0104	0.00306	0.000784
cg25214158	10	1.01E+08			-0.0167	0.00494	0.000785
cg06114363	1	26699448	*NF683;ZNF68*	S200;TSS200	0.00914	0.00269	0.000786
cg18998543	7	2769865	*GNA12*	3UTR	0.00663	0.00196	0.000787
cg07645542	19	19972043			0.0103	0.00302	0.000789
cg14169823	9	35560087	*RUSC2;RUSC2*	Body;Body	0.00752	0.00222	0.00079
cg06821959	9	95571711	*ANKRD19*	TSS200	0.0108	0.00319	0.00079
cg26253663	22	25799504			-0.022	0.00647	0.000792
cg11934419	6	1.13E+08	*A4;LAMA4;LA*	0;TSS200;TSS200;TSS2	-0.0126	0.00371	0.000792
cg08015278	3	73976278			0.0302	0.00892	0.000792
cg10275272	7	4784769	*FOXK1*	Body	0.00397	0.00117	0.000793
cg24501230	1	9212754	*MIR34A*	TSS1500	-0.00924	0.00273	0.000794

cg15209710	1	1.83E+08			0.0113	0.00333	0.000797
cg04640796	11	71791545	*T;LRTOMT;LI*	200;TSS200;1stExon;5U	-0.00154	0.000456	0.000797
cg03152353	9	1.39E+08	*NOTCH1*	Body	-0.0112	0.00331	0.000798
cg08955995	19	42503412	*GRIK5*	Body	0.0251	0.0074	0.000803
cg05507832	12	56727806	*N2;PAN2;PAI*	500;5UTR;5UTR;1stExo	-0.0112	0.0033	0.000803
cg06422693	4	48833587	*CIAD1;OCIAD*	5UTR;5UTR;5UTR;5UTI	0.00216	0.000638	0.000804
cg24804782	6	1.64E+08			-0.012	0.00356	0.000806
cg01024444	19	52222446	*HAS1*	Body	0.0376	0.0111	0.000807
cg11809342	2	1.6E+08	*TANC1;TANC1*	Body;Body	-0.00774	0.00229	0.000809
cg12344862	2	87019846	*;CD8A;CD8A;*	500;TSS1500;TSS1500	-0.0108	0.00319	0.000814
cg19472759	11	69456837	*CCND1*	Body	-0.00274	0.000809	0.000815
cg14963217	19	313076	*MIER2*	Body	-0.0066	0.00195	0.000815
cg20525037	3	64203257	*PRICKLE2*	5UTR	0.0106	0.00314	0.000819
cg02493846	1	1.52E+08	*OC10013211.*	TSS1500	0.0137	0.00406	0.000824
cg18943812	2	1682348	*PXDN*	Body	0.00568	0.00168	0.000827
cg01933079	5	65929713	*MAST4;MAST4*	Body;Body	0.0175	0.00517	0.000827
cg04155862	3	1.28E+08	*GLL;MGLL;MG*	R;5UTR;1stExon	-0.00805	0.00238	0.000827
cg08450404	19	58326441	*ZNF552*	TSS200	-0.00832	0.00247	0.00083
cg05038977	17	7476167	*EIF4A1*	1stExon	0.00343	0.00102	0.00083
cg09406795	11	64019655	*PLCB3*	Body	-0.0122	0.00362	0.000831
cg01199327	11	44626750	*CD82;CD82*	Body;Body	0.0139	0.00411	0.000833
cg13248574	14	77504561			0.0105	0.0031	0.000835
cg26819783	2	2.41E+08			0.00684	0.00203	0.000835
cg19260237	7	1282067			0.0156	0.00461	0.000835
cg11438134	21	46269016			0.0162	0.00479	0.000835
cg09844399	14	25526434			-0.00902	0.00267	0.000836
cg16738940	10	92575878	*TR7;HTR7;HTI*	dy;Body;Body	-0.0079	0.00234	0.000842
cg27283345	6	10416531	*FAP2A;TFAP2*	ody;TSS1500	-0.0119	0.00354	0.000842
cg03732014	21	30676184	*H1;BACH1;BA*	R;TSS1500;5UTR	-0.0112	0.00333	0.000844
cg03464560	2	2.19E+08	*CXCR2;CXCR2*	1500;TSS1500	-0.0102	0.00304	0.000845
cg16160720	5	86563744	*RASA1;RASA1*	1500;TSS1500	0.00239	0.000709	0.000846
cg01870519	6	693728	*EXOC2*	TSS1500	-0.0119	0.00353	0.000848
cg09270346	19	18060024			-0.0096	0.00285	0.000848
cg05519436	8	1.34E+08	*PHF20L1*	3UTR	0.00856	0.00254	0.00085
cg21105227	19	35633669	*D7;FXYD1;FX*	500;3UTR;3UTR	-0.00508	0.00151	0.000853
cg00218103	22	46465977			-0.008	0.00238	0.000855
cg16182465	11	76371946	*RRC32;LRRC3*	Body;Body	0.00692	0.00206	0.000856
cg13875287	2	64683186	*HSPC159*	Body	-0.0176	0.00522	0.000857
cg04347414	1	2084519	*CZ;PRKCZ;PR*	dy;Body;Body	0.024	0.00713	0.000858
cg19101326	8	1.06E+08	*ZFPM2;ZFPM2*	UTR;1stExon	-0.0103	0.00307	0.000859
cg11445760	11	64546351	*F1;SF1;SF1;SF*	S200;TSS200;TSS200	-0.00211	0.000628	0.000861
cg23227621	5	1.35E+08			-0.00655	0.00195	0.000866
cg22336034	22	30821633	*MTP18;MTP18*	S200;TSS200	-0.0032	0.000951	0.000866
cg13466917	17	57116416	*RIM37;TRIM3*	Body;Body	0.01	0.00299	0.000866
cg14042308	11	66104468	*RIN1*	TSS1500	-0.0112	0.00334	0.000867
cg21189321	3	9970062	*RC;IL17RC;IL1*	dy;Body;Body	-0.00457	0.00136	0.00087
cg12839593	14	61116227	*SIX1*	TSS200	0.00751	0.00224	0.000872

cg14647287	4	6927183	*C1D14;TBC1D*	Body;Body	-0.00769	0.00229	0.000873
cg05393741	12	1.07E+08	*RFX4*	Body	0.0207	0.00615	0.000874
cg11900742	8	1.14E+08	*D3;CSMD3;CS*	dy;Body;Body	-0.0144	0.0043	0.000874
cg09873933	5	1.35E+08	*TGFBI*	TSS200	-0.021	0.00625	0.000874
cg04857792	3	1.19E+08	*UPK1B*	5UTR	-0.0145	0.00432	0.000875
cg23570810	11	315102	*IFITM1*	Body	-0.0385	0.0115	0.000875
cg16018302	10	1.28E+08	*AM12;ADAM*	Body;Body	-0.00809	0.00241	0.000876
cg07806361	3	54331304	*CACNA2D3*	Body	-0.02	0.00596	0.000877
cg02699834	12	53039430	*KRT2*	Body	0.0122	0.00363	0.000879
cg13554849	9	96271203	*FAM120A*	Body	-0.00851	0.00253	0.000882
cg15883382	10	1.35E+08			-0.0046	0.00137	0.000883
cg00215133	18	76616808			0.0128	0.00381	0.000885
cg12218991	4	1.52E+08	*19;SH3D19;SH*	TR;3UTR;3UTR	0.0141	0.00421	0.000885
cg15182790	17	79318146			0.0113	0.00336	0.000885
cg22127703	7	91570873	*AKAP9;AKAP9*	Body;Body	0.00477	0.00142	0.000885
cg11769382	17	73851439	*WBP2;WBP2*	UTR;1stExon	-0.00432	0.00129	0.000886
cg26351104	1	51796558	*TTC39A*	Body	-0.00884	0.00264	0.000889
ch.16.1357280F	16	61883344	*CDH8*	Body	0.00302	0.000899	0.000889
cg02335185	20	60491905	*CDH4*	Body	0.0109	0.00325	0.000891
cg09580859	8	1.45E+08	*ZC3H3*	Body	-0.00367	0.00109	0.000893
cg00435616	7	1080473	*C7orf50*	Body	-0.0167	0.00499	0.000894
cg12573499	14	1.02E+08	*MIR134*	TSS1500;	0.00592	0.00177	0.000903
cg14663264	1	91192798			-0.011	0.00328	0.000903
cg20972294	11	1.33E+08	*OPCML*	Body	-0.014	0.00417	0.000904
cg13484901	12	14607340	*ATF7IP*	Body	0.0111	0.00331	0.000904
cg24060683	19	57755024	*NF805;ZNF80*	Body;5UTR	0.00655	0.00196	0.000906
cg24702826	6	86171768	*NT5E*	Body	0.0174	0.00518	0.000906
cg24807354	19	10629035	*S1PR5;S1PR5*	1500;TSS1500	0.00465	0.00139	0.000907
cg09996325	13	74599636	*KLF12*	5UTR	-0.0107	0.0032	0.000912
cg24158160	1	1.52E+08	*C1orf230*	TSS1500	-0.012	0.00358	0.000913
cg16137949	2	945423	*SNTG2*	TSS1500	-0.0103	0.00308	0.000915
cg25472458	7	50800607	*10;GRB10;GR*	5UTR	0.0126	0.00377	0.000915
cg12629349	2	54953117	*EML6*	Body	-0.00815	0.00244	0.000917
cg07048044	7	23470153	*IGF2BP3*	Body	0.0173	0.00516	0.00092
cg24957628	11	78664909	*ODZ4*	Body	-0.0117	0.00349	0.000921
cg05409693	12	78680140			-0.0203	0.00606	0.000921
cg07150062	14	1.05E+08	*ASPG*	TSS200	-0.0391	0.0117	0.000923
cg01080902	19	44039870	*ZNF575*	3UTR	0.0164	0.00491	0.000924
cg05775067	7	30602982			-0.00715	0.00214	0.000925
cg16000989	4	41983716	*AF4L1;DCAF4*	UTR;1stExon	0.0193	0.00578	0.000927
cg00361495	7	28995458	*TRIL*	1stExon	0.00667	0.00199	0.000931
cg23348789	17	28092503	*SSH2*	Body	-0.00777	0.00233	0.000933
cg21971621	11	65600773	*SNX32*	TSS1500	-0.00888	0.00266	0.000933
cg08365738	3	1.12E+08	*CDC80;CCDC8*	Body;Body	0.0171	0.00511	0.000935
cg16363586	19	17516329	*BST2*	1stExon	-0.0106	0.00316	0.000939
cg01289541	3	1.7E+08	*SLC7A14*	5UTR	0.014	0.0042	0.00094
cg25003777	6	2841626	*SERPINB1*	5UTR	0.00305	0.000912	0.000941

cg22853371	10	54068630			-0.00553	0.00165	0.000941
cg24283836	13	1.11E+08	*COL4A2*	Body	0.011	0.0033	0.000942
cg21742975	15	39169233			-0.0105	0.00315	0.000943
cg23641672	1	1.74E+08	*RABGAP1L*	Body	0.00449	0.00134	0.000945
cg07147449	12	1.15E+08			0.00799	0.00239	0.000946
cg05626226	4	1.07E+08	*FLJ20184*	Body	0.0208	0.00622	0.000946
cg20716058	7	1.51E+08	*i2;PRKAG2;PI*	y;TSS1500;Body	-0.00403	0.00121	0.000946
cg21366673	6	30459512	*HLA-E*	Body	-0.0157	0.00469	0.000947
cg08894020	5	1.55E+08			-0.0235	0.00705	0.000948
cg01797727	2	2.2E+08	*CYP27A1*	TSS1500	-0.012	0.00361	0.00095
cg00537351	5	14339109	*TRIO*	Body	-0.0105	0.00315	0.000952
cg04872399	1	2.01E+08	*TNNT2;TNNT2*	on;1stExon;1stExon;5U	-0.0131	0.00393	0.000953
cg02510297	12	1.17E+08	*00173;NCRN*	Body;Body	-0.0117	0.00351	0.000954
cg09121720	5	1.41E+08	*FCHSD1*	Body	-0.0042	0.00126	0.000955
cg07784872	16	85687041	*40182;KIAA0*	Body;Body	-0.0177	0.00531	0.000959
cg05862039	2	1.74E+08	*PGEF4;RAPGE*	Body;Body	0.0149	0.00446	0.000959
cg13968390	2	1.09E+08	*LT1C2;SULT1*	1500;TSS1500	0.0433	0.013	0.000964
cg19446350	13	44004267	*ENOX1;ENOX*	5UTR;5UTR	0.00748	0.00225	0.000966
cg01730534	1	1.75E+08	*ACYBP;CACYB*	S1500;TSS200	0.00399	0.0012	0.000966
cg06424550	10	12808422	*MK1D;CAMK*	Body;Body	-0.00594	0.00178	0.000967
cg04125962	16	27476250	*GTF3C1*	Body	0.0112	0.00338	0.000968
cg06213287	5	96478495	*LIX1;LIX1*	stExon;5UTR	-0.0147	0.00441	0.000969
cg11931501	11	2309109			-0.0118	0.00354	0.000969
cg16508603	11	6633496	*TAF10*	TSS200	-0.00398	0.0012	0.000971
cg16962558	6	13512880			0.00943	0.00283	0.000974
cg22772747	7	27164285	*HOXA3*	5UTR	-0.00574	0.00172	0.000976
cg26370729	5	76115672	*F2RL1*	Body	-0.0129	0.00387	0.000978
cg02047889	14	35452176	*SRP54;SRP54*	TR;1stExon;1stExon	0.00188	0.000564	0.000979
cg01076485	3	1.72E+08			-0.00352	0.00106	0.000982
cg07667161	6	42847125	*RPL7L1*	TSS1500	-0.0058	0.00174	0.000984
cg15043057	1	41444788	*CTPS*	TSS1500	-0.0102	0.00307	0.000984
cg00319168	2	1.77E+08	*HOXD13*	1stExon	0.00766	0.0023	0.000985
cg19045191	11	34167855	*NAT10;NAT1*	3UTR;3UTR	0.0066	0.00198	0.000985
cg09901201	1	53515142	*;SCP2;SCP2;S*	;Body;Body;Body	0.00683	0.00205	0.000986
cg03000848	16	396164	*AXIN1;AXIN1*	Body;Body	0.0171	0.00513	0.000988
cg01709766	11	5640318	*6-TRIM34;TR*	ody;TSS1500	0.0131	0.00393	0.000989
cg05129050	1	7178026	*CAMTA1*	Body	-0.019	0.00572	0.000991
cg22752023	10	1E+08	*ROXD2;PYRO*	UTR;1stExon	0.00846	0.00254	0.000991
cg07525313	6	1.16E+08	*FRK*	3UTR	0.0703	0.0212	0.000992
cg10126234	14	1.04E+08	*ZFYVE21*	Body	0.00785	0.00236	0.000992
cg24941681	20	33759921	*PROCR;PROC*	UTR;1stExon	-0.00756	0.00227	0.000993
cg21240441	9	1.4E+08	*PTGDS*	Body	-0.00892	0.00268	0.000993
cg22485171	5	1.69E+08	*CCDC99*	TSS200	0.00311	0.000936	0.000995
cg04574090	11	74178749	*KCNE3*	TSS200	-0.00837	0.00252	0.000996
cg23795217	12	54812000	*ITGA5*	Body	0.00916	0.00276	0.000996
cg04143750	2	2.33E+08	*EFHD1;EFHD1*	SS1500;Body	0.00905	0.00272	0.000996

Supplementary Table 3 Demographic and clinical variables in a testing sample set (N=238)

Variable		Mean ± SD	N (%)
Age		49.08 ± 8.05	
HIV-positive			238 (100)
Sex (male)			230 (96.6)
Race	EA		58 (24.4)
	AA		152 (63.9)
	Other		28 (11.8)
Smoker			134 (56.3)
AUDI-C		3.76 ± 3.15	
IDU+/HCV+			46 (19.3)
CD4+		434 ± 300	
log10 VL		2.59 ± 1.18	
CD8+		0.17 ± 0.08	
natural killer cells		0.07 ± 0.06	
B cell		0.08 ± 0.05	
Monocyte		0.12 ± 0.04	
Granulocytes		0.54 ± 0.13	
